# A new method for alignment of LC-MALDI-TOF data

**DOI:** 10.1186/1477-5956-9-S1-S10

**Published:** 2011-10-14

**Authors:** Zhiqun Tang, Lihua Zhang, Amrita K Cheema, Habtom W Ressom

**Affiliations:** 1 Lombardi Comprehensive Cancer Center, Georgetown University, Washington, DC, USA; 2 Bioinformatics Institute, 30 Biopolis Street, Matrix, 138672 Singapore

## Abstract

**Background:**

In proteomics studies, liquid chromatography coupled to mass spectrometry (LC-MS) has proven to be a powerful technology to investigate differential expression of proteins/peptides that are characterized by their peak intensities, mass-to-charge ratio (*m/z*), and retention time (*RT*). The variable complexity of peptide mixtures and occasional drifts lead to substantial variations in *m/z* and *RT* dimensions. Thus, label-free differential protein expression studies by LC-MS technology require alignment with respect to both *RT* and *m/z* to ensure that same proteins/peptides are compared from multiple runs.

**Methods:**

In this study, we propose a new strategy to align LC-MALDI-TOF data by combining quality threshold cluster analysis and support vector regression. Our method performs alignment on the basis of measurements in three dimensions (*RT*, *m/z*, intensity).

**Results and conclusions:**

We demonstrate the suitability of our proposed method for alignment of LC-MALDI-TOF data through a previously published spike-in dataset and a new in-house generated spike-in dataset. A comparison of our method with other methods that utilize only *RT* and *m/z* dimensions reveals that the use of intensity measurements enhances alignment performance.

## Introduction

Proteomics has been extensively used to study the protein expression of cells under various physiological conditions. As an indispensable tool for proteomics, mass spectrometry (MS) can identify and determine the abundance of various proteins or peptides in easily accessible, non-invasive body fluid samples such as serum and urine. However, the presence of a large number of peptides or proteins and their dynamic range in the body fluid raise many challenges in the data analysis. A separation procedure prior to MS analysis has been utilized to reduce the complexity of samples. Liquid chromatography (LC) coupled with MS (LC-MS) has become a common platform in proteomics studies, since it combines the physical separation capabilities of LC with the mass analysis capabilities of MS of protein mixtures.

LC-MS characterizes peptides and proteins by their retention time (*RT*) and mass-to-charge ratio (*m/z*), and quantifies them with peak intensity (e.g., ion count). Comparison of multiple LC-MS runs generated from different conditions (such as healthy and diseased) provides a comprehensive quantitative overview of thousands of peptide concentration among samples to identify differentially abundant peptides. One of the most significant challenges in label-free comparison of multiple LC-MS runs is to ensure that the peptide peaks are aligned correctly prior to identification of differential abundance. Without alignment, the same peptide may have different *m/z* and *RT* values across multiple runs due to *m/z* and *RT* drifts. The shifts in the *m/z* dimension typically have small deviation, but the *RT* dimension is prone to large and non-linear shifts due to the LC instrument conditions, peptide-peptide interactions, and peptide/protein modifications. Improper correction of *m/z* and *RT* shifts may lead to false discovery of biomarker candidates. Therefore, a reliable peak alignment strategy is required prior to comparing biological groups represented by multiple LC-MS runs.

Various alignment methods have been reported including correlated optimized warping (COW) [1,2], dynamic time warping (DTW) [2], and continuous profile model (CPM) [3]. These methods perform alignment on the basis of raw LC-MS data or total ion chromatograms (TIC). The latter is obtained by calculating the sum of the ion abundances across the *m/z* dimension for each *RT* point. Due to the enormous size of the data, alignment methods that are based on raw LC-MS data need large memory and are computationally demanding. Since the comparison of many samples is common in the biomarker discovery studies, more efficient alignment methods are needed. While TIC-based alignment methods address this concern, they ignore the *m/z* dimension, which might contain relevant information on the *RT* deviation [4]. Moreover, peptides with different *m/z* values may have different *RT*. For example, two peptides eluting at the same time in one experiment may elute at different time in another experiment, and the elution order of these peptides may vary from one experiment to another. TIC-based alignment methods cannot capture such variability. Alignment methods, which use peaks detected from the raw data involving both *m/z* and *RT* dimensions offer a better solution than TIC-based methods in addressing the nonlinear *RT* shifts.

Various peak-based alignment methods have been reported in the literature [5-7]. In these methods, the relevant peaks are extracted from raw data and the information contained in the peaks is applied for alignment. Since the number of the peaks is much smaller than that of the raw LC-MS data, these methods need less computer memory and shorter computation time. Thus, they are suitable for alignment of a large number of LC-MS runs. As one of the most frequently used methods, hierarchical clustering was first applied for alignment of spectra obtained from matrix-assisted laser desorption-ionization time-of-flight (MALDI-TOF) mass spectrometer [8]. The method was later extended for alignment of LC-MS runs [6,7]. Tibshirani et al. [8] obtained peaks from individual MALDI-TOF spectra and applied the complete linkage hierarchical clustering method to align peaks across experiments. They defined distance metrics between peaks as Euclidean distance in *m/z* dimension. Once clustering was completed, the dendrogram was cut into clusters, and all peaks belonging to a cluster were considered to be the same peak. A “consensus” peak was extracted by taking the mean *m/z* value of each cluster. Jaitly et al. [6] utilized the complete- and the single-linkage clustering approaches to search for feature clusters across multiple LC-MS runs. Podwojski et al. [7] employed a hierarchical average-linkage cluster analysis on LC-MS runs to derive “well-behaved” groups with which they estimated and corrected the *RT* deviation for each run with regression methods. Lange et al. [5] described a pose clustering method by which peak-picked LC-MS datasets were transformed with an affine map, and used the parameters of the transformation to align the datasets.

Most clustering methods typically involve the following steps in peak-based spectral alignment: (1) identify “high intensity” peaks; (2) cluster these peaks; (3) align the peaks with respect to the cluster center; and (4) use regression or other mathematical model to estimate shifts of all peaks and to correct the shifts.

There are some drawbacks in using the above-mentioned clustering-based approach for LC-MS alignment. For instance, most clustering methods only consider *m/z* and *RT* dimensions, ignoring peak intensity measurements that might be important to accurately align LC-MS runs. In addition, it is important to find a suitable threshold to cut the dendrogram tree or decide the number of clusters. Accurate estimation of the number of clusters is desired, but it is clearly a challenging task.

In this study, we propose a new LC-MS alignment method based on cluster analysis of peaks. We define a distance metrics that involves *m/z*, *RT*, and ion intensity measurements to perform clustering. Specifically, we use a partition-based clustering approach to find clusters by evaluating the quality of clusters and a non-linear regression to correct *RT* shifts. We evaluate the performance of this method through two spike-in datasets, an in-house generated dataset and a previously published dataset.

## Materials and methods

### LC-MALDI-TOF datasets

#### In-house dataset

This dataset consists of 12 LC-MALDI-TOF runs derived from two groups of samples: (1) serum samples without spike-in peptides, and (2) serum samples spiked with a mixture of peptides containing 40 fmol/μl of angiotensin (1296.70 Da), 26 fmol/μl of Glu1-Fibrinopeptide B (1570.70 Da), 40 fmol/μl of ACTH 1-17 fragment (2093.10 Da), and 30 fmol/μl of ACTH 18–39 fragment (2465.19 Da). Each group is comprised of three experimental replicates with each replicate measured in duplicate.

Blood sample of a healthy volunteer was collected by a trained phlebotomist, and was processed to enrich native peptides in the low molecular weight fraction of serum. Forty-five microlitre of serum sample was desalted on C8 magnetic beads and ultrafiltered in 25% acetonitrile on 50 kDa Microcon membranes.

Peptide separation was performed on a Tempo nano MDLC system (Applied Biosystems/MDS Sciex, Toronto, Canada) in conjunction with a Probot MALDI spotting device (Dionex-LC Packings). We concentrated 5μl of sample on a 0.5 cm x 300 μm i.d. C18 trap column using mobile phase A (2% acetonitrile, 0.1% TFA) delivered at 20 μl/min. The sample was then eluted onto a 150 mm x 75 μm i.d Vydac C18 column (5μm particular size, 300Å pore size, Grace Vydac, Hesperia, CA) using a linear gradient of solvent B (98% acetonitrile, 0.1% TFA ) in solvent A as follows: from 5% to 60% of B in 180 min, switched to 95% solvent B for 10 min, followed by 20 min re-equilibration with solvent A at a constant flow rate of 0.3 μl/min. The elution of the column was mixed with a solution of 3.75 mg/ml α-cyano-4-hydroxycinnamic acid MALDI matrix (α-CHCA) (ACROS Organics, Geel, Belgium) at the exit. The CHCA was prepared daily and delivered at a flow rate of 0.6 μl/min. The mixture of elution and CHCA was spotted into the MALDI plate through the Probot spotting system. Each spot represented a one-minute "fraction" (0.9 μl) of the reverse phase gradient. Triplicate runs of the sample, each containing 192 spots, were distributed on the MALDI plates.

The spots representing chromatographic fractions were analyzed using ABI 4800 MALDI-TOF/TOF, equipped with a neodymium: yttrium-aluminum-garnet laser emitting at λ = 355 nm with a repetition rate of 200 Hz. An average of 50 shots at each of 20 positions was collected for a total of 1000 shots/spot in the reflector positive mode in the mass range of 800-5000 Da. Each MALDI plate was measured in duplicate.

#### Benkali’s dataset [**9**]

This dataset consists of 24 LC-MALDI-TOF runs, which were obtained from urine samples of three healthy volunteers. Aliquots of each sample were spiked with three peptides at four different concentrations (0X, 0.25X, 0.5X and 1X), where 1X = 120 fmol/μl of human angiotensin II (1046.54 Da), 80 fmol/μl of neurotensin (1672.91 Da), 100 fmol/μl of ACTH 18–39 fragment (2465.19 Da). Each spike-in sample was measured in duplicate by off-line coupling of Ultimate 3000 nano-HPLC system and ABI 4800 MALDI-TOF/TOF. Each LC-MALDI-TOF run contained 358 fractions. Each fraction represented a 12-second interval (containing 15 μl of urine elution and 45 μl of α-CHCA matrix) of the LC gradient.

### Data preprocessing

In both datasets, all ions with *m/z* below 1000 were excluded. We performed binning with a bin size of 200 ppm, baseline correction by using the loess method, normalization by dividing each spectrum by its total ion count, denoising using wavelet shrinkage, and peak detection by finding local maxima of the denoised data. These preprocessing steps were applied at MALDI-TOF spectrum level. Furthermore, the peak intensities for each LC-MALDI-TOF run were normalized to the median intensity of one reference MALDI-TOF spectrum, which has the highest median intensity compared to the rest of spectra. The normalized peaks from all runs were combined together in three dimensions (*m/z*, *RT*, and ion intensity).

### Alignment algorithm

#### QT clustering

Previously applied to identify coexpressed genes from microarray data, quality threshold (QT) clustering method was originally described by Heyer et al [10]. It is based on the unique constraint of the cluster diameter, as a user-defined parameter. This method has no other requirement of dividing data into a predetermined number of clusters, while this requirement must be satisfied by other clustering algorithms such as k-means.

Prior to the QT cluster analysis, for each LC-MALDI-TOF run, we selected the peak candidates with intensity higher than the mean intensity of that specific run. The candidates from all runs were combined together to form a peaklist, and the peaklist was used in cluster analysis procedure. The QT clustering is an iterative algorithm and starts with a global set that includes all elements (peaks) of the dataset (peaklist), and returns a set of clusters that satisfies the quality threshold. Such threshold is defined in terms of cluster diameter. The algorithm includes the following steps:

a) For each element, a candidate cluster seeded by this element is formed.

b) Such cluster is iteratively added by element, which can minimize the increase in cluster diameter. This process continues until no element can be added without surpassing the diameter threshold.

c) Once a cluster is generated, all elements within this cluster are excluded from the subsequent analysis.

d) Repeat Steps a–c for the subsequent cluster identification, until all elements belong to a particular cluster.

e) Clusters with fewer elements than a specific number are removed, and the elements (peaks) in these clusters are excluded. Other clusters are retained as qualified clusters.

f) Iterate Steps a-e multiple times, each time stating from different candidate cluster seeds of the first element. The summation of the distances within the qualified clusters is used to compare different iterations. The iteration with the smallest summation of within-clusters is our solution and the qualified cluster information in this iteration is used in the regression analysis.

The distance between peak_1_ and peak_2_ (*d*_1,2_) in the cluster analysis is a weighted Euclidean distance in the *m/z*, *RT* and intensity (*int*) dimensions, i.e.,  where *w_mz_*, *w_RT_*, *w_int_* are weights associated with the three dimensions.

The *RT*, *m/z*, and log transformed intensity were standardized to have zero mean and unit standard deviation. *w_RT_* was set to one. Considering the measurement precision range of mass spectrometry and HPLC, the penalty for the *m/z* change should be much higher than the penalty for the *RT* change. Therefore, *w_mz_* was screened between 100 to 2000. In most cases, the penalty for the *m/z* change should be higher than the penalty for the intensity change. We screened *w_int_* between 0.1 to *w_mz_*/2. The minimum number of peaks within one cluster was set to two. The maximum cluster diameter was optimized along with the optimization of weight values (*w_mz_*, *w_int_*) of the distance metric. A criterion used to screen the parameters was the ratio of the number of “good-behaved” clusters to the total number of clusters. The behavior of a cluster is defined as the ratio of the number of all runs to the number of replicate runs within this cluster.

#### Support vector regression

Following clustering, for each LC-MALDI-TOF run, a support vector regression (SVR) was applied to model *RT* deviation and to correct the shifts of all detected peaks. SVR maps the data into a higher-dimensional feature space via a non-linear kernel function, and a linear regression is conducted in this feature space [11]. Let the training data be {(*x_1_*, *y_1_*),….(*x_l_*, *y_l_*)}, where *x* represents (*m/z*, *RT*, *intensity*)*^T^* and *y* denotes the average *RT* values with clusters, the SVR model *f*(*x*,*w*) will be given by

Where *K* is a kernel function denoting a set of transformation (e.g.., we used a. Gaussian kernel), and *b* is the “bias”. SVR performs regression in the high-dimension feature space using ε-insensitive loss, and simultaneously reduce the model complexity by minimizing the Euclidean norm ||*w*||. This problem can be written as a convex optimization problem by minimizing 0.5||*w*|| subject to

This optimization problem can be transformed into the dual problem and its solution given by

The coefficients α, α*, and *b* are determined by maximizing the following Lagrangian expression

under the following conditions of

## Results

### In-house dataset

**Figure 1:**
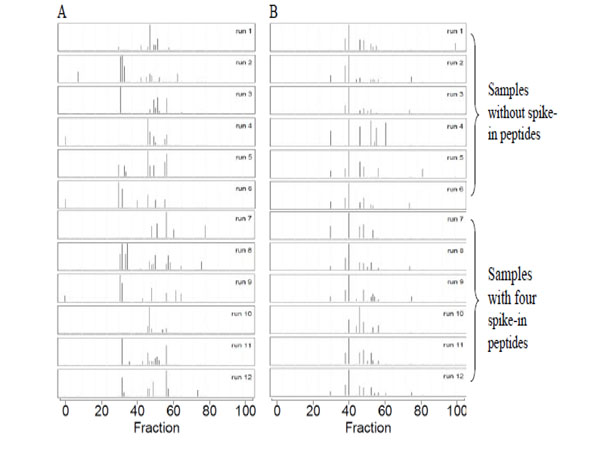
**The TICs of LC-MALDI-TOF maps of 12 serum samples from our in-house dataset before (Panel A) and after alignment (Panel B).*** x* axis is the fraction number from 1 to 100 and *y* axis is the TIC values calculated from *m/z* between 1510 to 1515 Da. Runs 1 to 6 were obtained from the serum samples without spike-in peptides and Runs 7 to 12 were obtained from serum samples with four spike-in peptides.

We evaluated our alignment algorithm with our in-house LC-MALDI-TOF dataset, which contained two groups of samples, (1) serum samples without spike-in peptides and (2) serum samples spiked with four peptides. All signals with *m/z* < 1000 Da were excluded since matrix had negative impacts on signal peaks with mass below 1000. The alignment procedure was performed on 12 runs, 6 runs in each group. Table 1 gives the coefficients of variation (CVs) and the Pearson correlation coefficients calculated for our in-house LC-MALDI-TOF dataset before and after alignment for all peaks across the *m/z* ranging from 1000 to 5000. For samples without spike-in peptides, the average CV was decreased from 28% to 22%, and the average Pearson correlation coefficient was increased from 0.65 to 0.94. For samples with spike-in peptides, the average CV was decreased from 40% to 22%, and the average Pearson correlation coefficient was increased from 0.79 to 0.95. Fig. 1 depicts TICs of LC-MALDI-TOF maps of 12 serum samples before and after alignment. As shown in figure 1, our alignment method has reduced the misalignment problem observed in the raw LC-MALDI-TOF data. Table 2 shows the *m/z* and *RT* values of the four spike-in peptides before and after alignment.

**Table 1:** The CV and correlation of our in-house LC-MALDI-TOF dataset before and after alignment. The result of the best performing method is in boldface.

			After alignment							
		
	Original		Our method using three dimensions (*RT*, *m/z*, intensity)		Our method using two dimensions (*RT*, *m/z*)		Podwojski’s method (hierarchical clustering and linear regression)		Podwojski’s method (hierarchical clustering and loess)	
	
Group	CV	Corr	CV	Corr	CV	Corr	CV	Corr	CV	Corr
1	32	0.31	**20**	**0.89**	31	0.71	26	0.68	29	0.79
2	30	0.39	**15**	**0.9**	26	0.82	19	0.86	29	0.89
3	28	0.45	**17**	**0.89**	31	0.7	22	0.67	30	0.71
4	27	0.5	**21**	0.93	26	0.83	25	0.94	24	**0.97**
5	33	0.36	**25**	**0.83**	31	0.75	26	0.77	28	**0.83**

CV=coefficient of variation (%); Corr=Pearson correlation coefficient; Group 1=samples without spike-in peptides; Group 2=samples with spike-in peptides; Group 3=Groups 1 and 2 combined.

**Table 2:** The *m/z* and *RT* values of the spike-in peptides in our in-house LC-MALDI-TOF dataset before and after alignment. The ranges of *RT* before alignment were obtained from the MALDI-TOF-TOF spectra of the samples with spike-in peptides.

	Original (before alignment)		After alignment	
	
Spiked-in peptides	*m/z* (Dalton)	*RT* (fraction)	*m/z* (Dalton)	*RT* (fraction)
Angiotensin	1296.7	41-44	1296.03	52
Glu1-Fibrinopeptide B	1570.7	31-34	1570.65	35
ACTH 1-17 fragment	2093.1	22-24	2093.97	30
ACTH 18–39 fragment	2465.2	55-60	2465.13	63

### Benkali’s dataset

**Figure 2:**
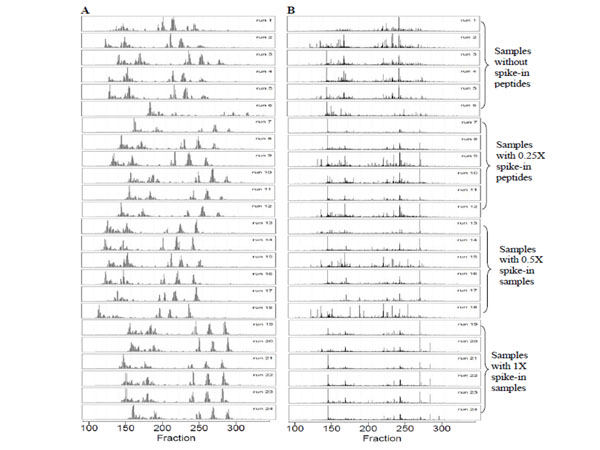
**The TICs of LC-MALDI-TOF maps of 24 urine samples from Benkali’s dataset before (Panel A) and after (Panel B) alignment.*** x* axis is the fraction number from 100 to 358 and *y* axis is the TIC values calculated from *m/z* 2000 to 2500 Da. Runs 1 to 6 were obtained from urine samples without spike-in peptides. Runs 7 to 12 were obtained from urine samples with 0.25X spike-in peptides. Runs 13-18 were obtained from samples with 0.5X spike-in peptides. Runs 19-24 were obtained from samples with 1X spike-in peptides.

We performed additional evaluation of our algorithm using the Benkali’s dataset [9], which represents four groups of samples: urine samples spiked with four different concentrations (0X, 0.25X, 0.5X and 1X) of peptides. Each group had six samples containing both biological replicates and experimental replicates. Fig. 2 depicts TICs of LC-MALDI-TOF maps of 24 urine samples before and after alignment. It is obvious that the dataset had a large *RT* shift before alignment. This *RT* shift could mask the relative peptides which introduce false biomarker discovery. As shown in Table 3, the average CV for each group was reduced from 27%-32% to 15%-21%, while the Pearson correlation coefficients of each group were increased from 0.31-0.50 to 0.89-0.93.

**Table 3:** The CV and correlation of the Benkali’s LC-MALDI-TOF dataset before and after alignment. The result of the best performing method is in boldface.

			After alignment							
		
	Original		Our method using three dimensions (*RT*, *m/z*, intensity)		Our method using two dimensions (*RT*, *m/z*)		Podwojski’s method (hierarchical clustering and linear regression)		Podwojski’s method (hierarchical clustering and loess)	
	
Group	CV	Corr	CV	Corr	CV	Corr	CV	Corr	CV	Corr
1	32	0.31	**20**	**0.89**	31	0.71	26	0.68	29	0.79
2	30	0.39	**15**	**0.90**	26	0.82	19	0.86	29	0.89
3	28	0.45	**17**	**0.89**	31	0.7	22	0.67	30	0.71
4	27	0.5	**21**	0.93	26	0.83	25	0.94	24	**0.97**
5	33	0.36	**25**	**0.83**	31	0.75	26	0.77	28	**0.83**

CV=coefficients of variation (%); Corr=Pearson correlation coefficient; Group 1=samples without spike-in peptides; Group 2=samples with 0.25X spike-in peptides; group 3: samples with 0.5X spike-in peptides; group 4: samples with 1X spike-in peptides; Group 5: Group 1-4 combined.

## Discussion

We introduce a new method that utilizes the clustering concept to correct *RT* shifts in LC-MALDI-TOF data. Currently, the alignment of liquid chromatography-electrospray ionization mass spectrometry (LC-ESI-MS) runs is well discussed in the literature [3,12-14]. However, more research is needed to build alignment methods specifically targeted to LC-MALDI-TOF runs. Since there are some differences between the two systems, several concerns need to be addressed to align LC-MALDI-TOF runs. First, in LC-ESI-MS, the on-line coupling technique forms continuous maps, while in LC-MALDI-TOF the off-line coupling technique generates discrete maps [15]. To adjust *RT* shifts in LC-MALDI-TOF maps, a discrete alignment method is expected to be more efficient than continuous methods, which have been developed and extensively investigated in LC-ESI-MS data [15]. Although a reasonable approximation can be made by a continuous alignment method to solve the discrete alignment optimization problem, an assumption must be made that the elution order of peptides is conserved throughout the experiments [5,15]. Second, the reproducibility level in LC-MALDI-TOF runs is relatively poor, which results from the semi-quantitative nature of peak intensity due to sample preparation and matrix cocrystallization [16]. This is considered to be one of the major problems to align data in between the neighboring fractions. Third, single-charged protonated ions in LC-MALDI-TOF could significantly simplify the alignment procedure in dealing with the peak isotopic distributions compared with multiple charge ions in LC-ESI-MS system.

Taking the differences between LC-ESI-MS and LC-MADLI-TOF MS into consideration, in this paper we introduce a new peak alignment method for LC-MALDI-TOF data analysis. Our method uses QT cluster analysis to cluster the peaks based on *m/z*, *RT*, and intensity. Support vector regression is used to model and correct the *RT* shifts in the entire *m/z* and *RT* dimensions. Cluster analysis on the basis of peaks from LC-MALDI-TOF data allows us to determine clusters of peaks that potentially represent the same biological entities. Clustering method is especially suitable for alignment of LC-MALDI-TOF runs due to their high resolution and discrete property that allows us to easily breakdown the data into “peak” and “non-peak” regions compared with LC-ESI-MS runs.

Our method is evaluated through two datasets. The first dataset (in-house dataset) is generated by our group, containing experimental replicates. The CVs within specific groups are decreased from 28%-40% to 22%, and the correlation coefficients are increased from 0.65-0.79 to 0.94-0.95. The second dataset (Benkali’s dataset) is obtained from published literature containing biological replicated. The CVs within specific groups are decreased from 27%-33% to 15%-25%, and the correlation coefficients are increased from 0.31-0.50 to 0.89-0.93.

We compared the alignment results obtained by using three dimensions (*RT*, *m/z*, intensity) with those obtained from analysis of two dimensions (*RT* and *m/z*), only. In the in-house dataset, we observed that the use of three dimensions leads to significantly better correlation among samples that belong to the same group (Table 1). For the Benkali’s dataset, the CVs for all groups are significantly decreased, and the Pearson correlation coefficients are significantly increased with the addition of intensity (Table 3).

As an example for comparison, Podwojski’s methods [7] were applied to both datasets. Average-linkage cluster analysis and linear/loess regression methods with two dimensions (*RT* and *m/z*) were involved in those methods. The results are shown in Table 1 for the in-house dataset and in Table 3 for the Benkali’s dataset. The CVs obtained with our method are smaller than those with Podwojski’s methods in nearly all groups, with one exception (spike-in peptides in our in-house data set). The Pearson correlation coefficients with our method are larger than those with Podwojski’s methods in nearly all groups, with one exception (samples with 1X spike-in peptides in Benkali’s dataset). These results indicate that our alignment algorithm performs better than Podwojski’s methods. This is attributed to three aspects: (1) peak intensity is used in the distance metrics, (2) predefined number of clusters is not required in the QT clustering, and (3) non-linear kernel function is used in support vector regression. In the QT clustering, some peaks are allowed to fall out of any specific clusters, indicating that outlier and noisy data can be filtered during the clustering procedure. This is very useful when aligning LC-MALDI-TOF runs from different groups of samples. Compared with other clustering-based alignment methods, our QT clustering method has to screen three parameters (maximum cluster diameter, w_mz_, and w_int_), which increase the computational time. Future work will focus on addressing this limitation through optimization of the algorithm.

## Competing interests

No conflict interest is declared

## Authors' contributions

ZT: conception and conduct of experimental design, development of data analysis tools, writing and revision of manuscript, figure 2 and table preparation; LZ: source of proteomic data, manuscript critique and revision; AKC: manuscript critique and revision; HWR: conception of project, development of data analysis tools, writing and revision of manuscript. All authors read and approved the final manuscript.
